# NMR Spectroscopic Signatures of Cationic Surface Sites from Supported Coinage Metals Interacting with N-Heterocyclic Carbenes

**DOI:** 10.1021/jacs.4c00200

**Published:** 2024-03-01

**Authors:** Shahar Dery, Weicheng Cao, Chengbo Yao, Christophe Copéret

**Affiliations:** † Department of Chemistry and Applied Biosciences, ETH Zürich, CH-8093 Zürich, Switzerland

## Abstract

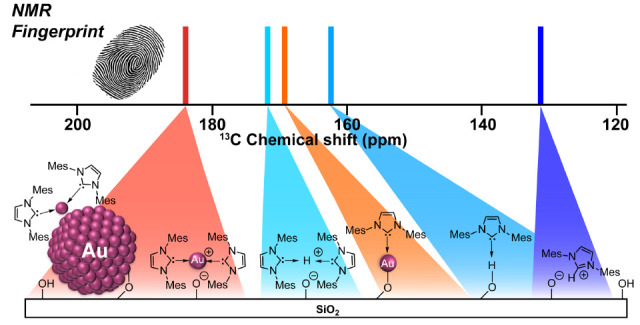

N-heterocyclic carbenes (NHCs) have been extensively studied to modulate the reactivity of molecular catalysts, colloids, and their supported analogues, being isolated sites, clusters, or nanoparticles. While the interaction of NHCs on metal surfaces has been discussed in great detail, showing specific coordination chemistry depending on the type of NHC ligands, much less is known when the metal is dispersed on oxide supports, as in heterogeneous catalysts. Herein, we study the interaction of NHC ligands with Au surface sites dispersed on silica, a nonreducible oxide support. We identify the easy formation of bis-NHC ligated Au(I) surface sites parallel to what is found on metallic Au surfaces. These species display a specific ^13^C NMR spectroscopic signature that clearly distinguishes them from the mono-NHC Au(I) surface sites or supported imidazoliums. We find that bis-ligated surface species are not unique to supported Au(I) species and are found for the corresponding Ag(I) and Cu(I) species, as well as for the isolobal surface silanols. Furthermore, the interaction of NHC ligand with silica-supported Au nanoparticles also yields bis-NHC ligated Au(I) surface sites, indicating that metal atoms can also be easily extracted from nanoparticles, further illustrating the dynamics of these systems and the overall favorable formation of such bis-ligated species across a range of systems, besides what has been found on crystalline metal facets.

Following pioneering works in molecular chemistry,^1^ N-heterocyclic carbenes (NHCs) were introduced in surface science and colloidal chemistry as molecular stabilizing ligands in the late 2000s, starting with coinage metals.^2−6^ Their popularity as surface ligands stems from their superior chemical tunability and robustness when exposed to various reaction conditions (thermal treatments, pH extremes, organic solvents), surpassing the classical thiol-based ligands.^7−10^ The interaction of NHCs with metal surfaces, e.g. Cu and Au, has thus been intensively investigated via surface science characterization tools such as scanning tunneling microscopy (STM), Raman spectroscopy, X-ray photoelectron, and absorption spectroscopy (XPS and XAS, respectively), often combined with computational studies.^6,11−20^ These studies focusing on well-defined group 11 metal crystalline facets have revealed various adsorption geometries on metal surface sites with specific binding energies.^11^ NHCs mainly adopt two stable adsorption modes on metal surfaces: (i) NHC ligand bound to one metal adatom and residing perpendicular to the surface (Figure 1a, center - mono-NHC metal adatom)^11,17,18,21^ and (ii) bis-NHC metal cationic species oriented horizontally with respect to the surface and resulting from the extraction of one surface metal site through a stabilization with two NHC ligands (Figure 1a, right - bis-NHC metal adatom).^14,18^ The formation of one or the other has been traced back to specific van der Waals interactions between the N-bound substituents of the NHC ligands and the metallic surface.^14,16^ NHCs with bulky aromatic substituents such as diisopropylphenyl (IDipp) or mesityl (IMes) favor the horizontal adsorption mode, maximizing the nonbonding π-interactions with the surface,^13,17^ whereas the use of NHCs with short aliphatic substituents such as methyl (IMe) and isopropyl (IiPr) favors the perpendicular orientation.^13,18^

**Figure 1:**
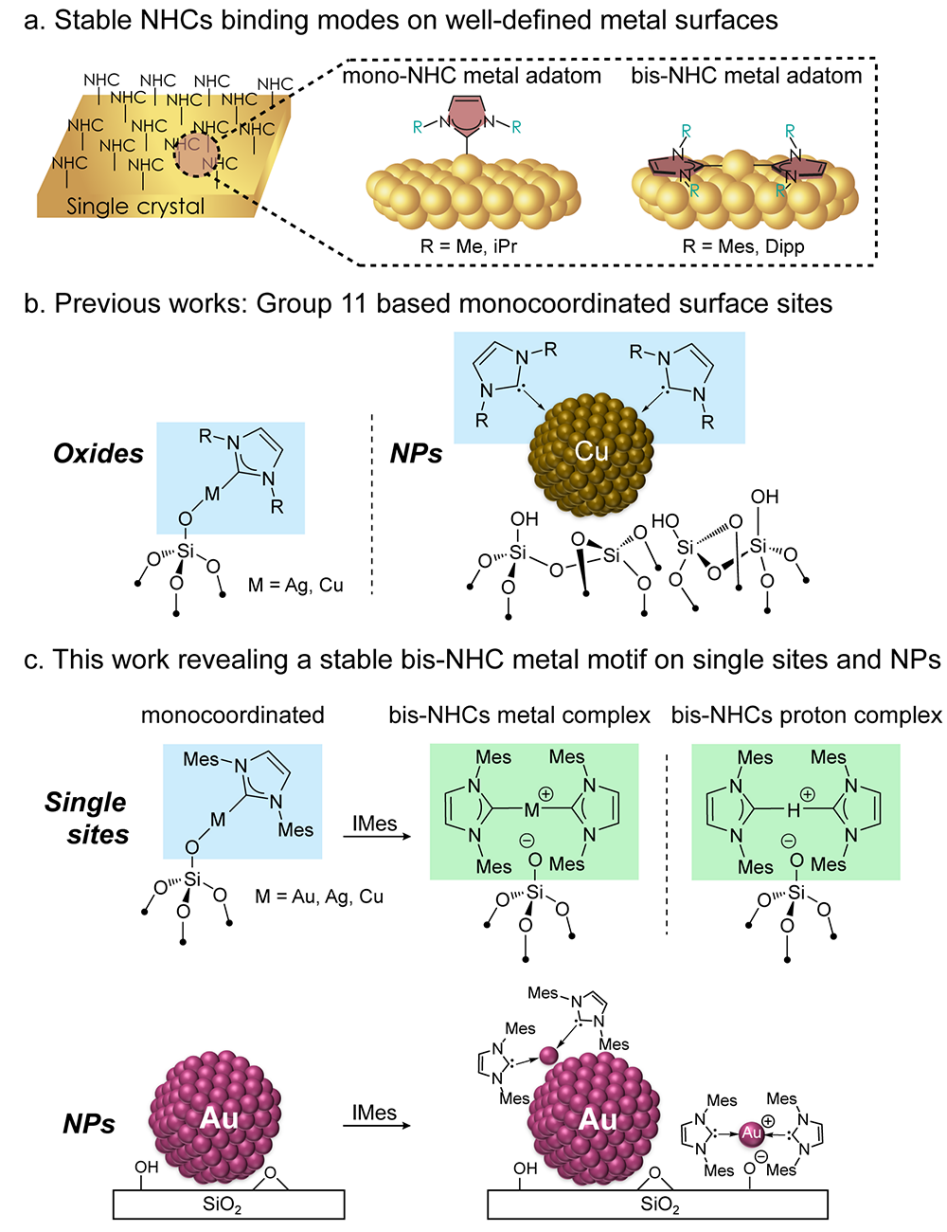
(a) Schematic illustration of the mono- and bis-NHCs binding modes on well-defined metal surfaces (b) Previous works demonstrating group 11-based surface sites: monocoordinated NHC-Ag and Cu complexes and the proposed monocoordination on silica-supported CuNPs (c) The preparation of a stable bis-NHC metal motif on oxide surfaces and its bis-NHC proton analog. The solid-state NMR signature of the bis-NHC Au motif on AuNPs/SiO_2_.

In parallel to these fundamental studies in surface science, NHCs have been introduced on both colloids and supported nanoparticles (NPs) to modulate their catalytic performances.^10,18,22−25^ In these reports, the binding of NHC ligands on the metal surface is proposed to adopt a vertical monocoordinated NHC configuration.^22,23^ Detailed nuclear magnetic resonance (NMR) spectroscopic investigations of silica-supported Cu species have shown that the spectroscopic signatures could in principle help to distinguish various surface sites, in particular, NHC adsorbed on isolated metal surface sites vs metallic surface sites from protonated imidazolium species (Figure 1b).^26^ Nonetheless, bis-NHC metal cationic surface sites have so far not been considered on coinage metal supported on oxide surfaces, raising questions about their existence and specific (NMR) spectroscopic signatures.

Herein, we explore the interaction of NHC ligands with Au surface sites dispersed on silica, a prototypical nonreducible oxide free of Lewis acid sites, starting with well-defined isolated Au(I) sites. By conducting solid-state ^13^C NMR spectroscopy measurements benchmarked with ^13^C labeled molecular models, we demonstrate the formation of mono- and bis-NHC Au(I) surface sites and unambiguously identify their specific NMR spectroscopic signatures. We show that these bis-ligated surface species can also be found for Ag(I) and Cu(I) sites, as well as for the isolobal protons. Finally, we show that silica-supported AuNPs postfunctionalized with NHC ligands also generate similar species, indicating that Au atoms of the corresponding supported nanoparticles can also be readily extracted, forming similar bis-ligated NHC Au(I) surface sites, further drawing a parallel with main concepts discussed in surface science. We first focus on generating well-defined isolated Au(I) surface species stabilized by a NHC ligand by grafting [Au(NHC)(Mes)] (NHC = IMes i.e. 1,3-Bis(2,4,6-trimethylphenyl)-1,3-dihydro-2*H*-imidazol-2-ylidene) on amorphous silica partially dehydroxylated at 700 °C (Figure 2a, SiO_2–700_; 0.35 mmol_OH_·g^–1^; ca. 1 OH per nm^2^). The IR spectrum shows a significant decrease in the OH band at 3743 cm^–1^ (Figure 2b, **1-Au**), consistent with grafting. This reactivity sharply contrasts with the absence of reactivity of Au_5_Mes_5_ toward silica.^27^ Monitoring grafting by solution ^1^H NMR shows that 0.45 equiv. of MesH is released per surface silanols, consistent with 45% grafting.

**Figure 2:**
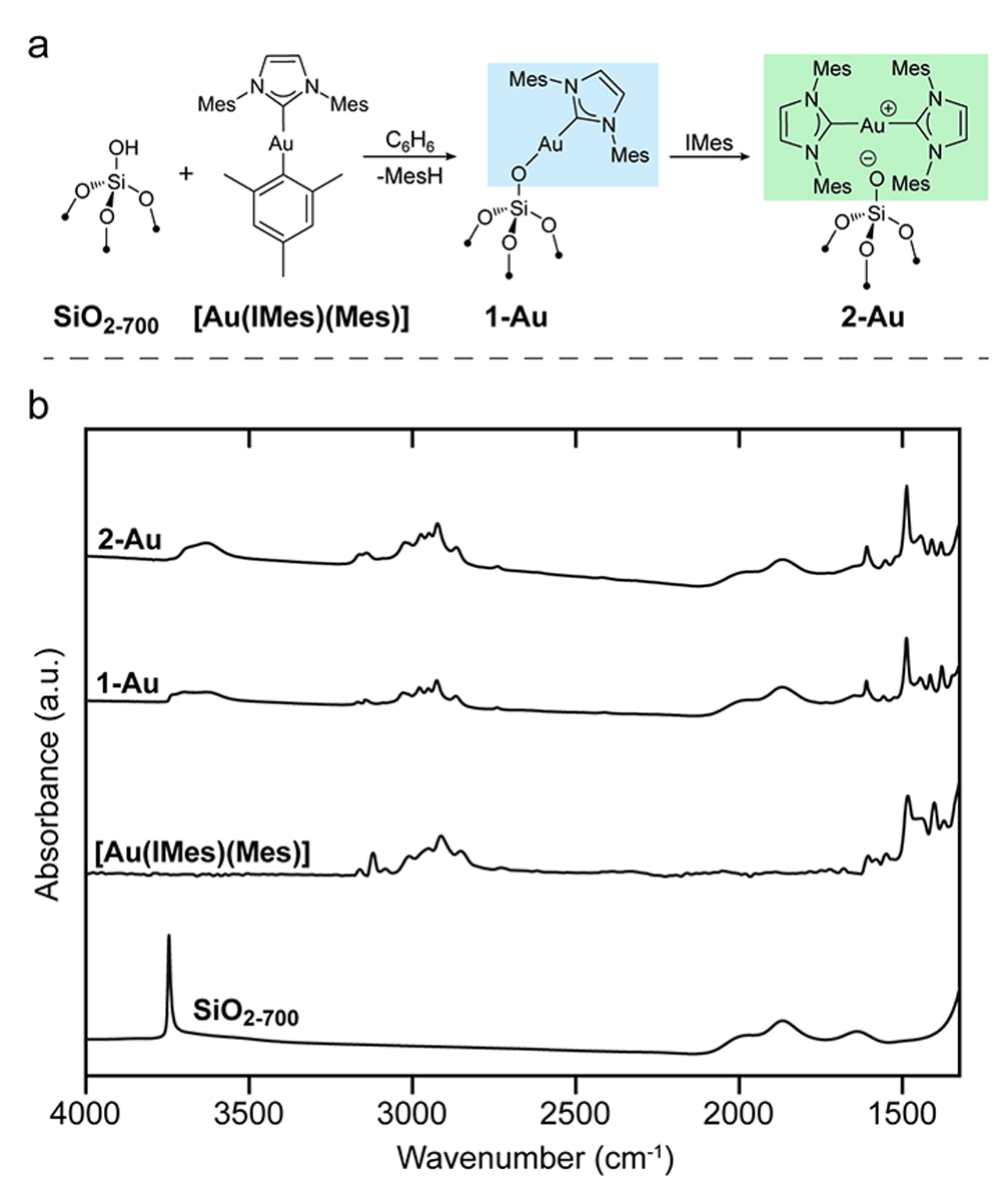
(a) Schematic illustration of the grafting procedure and preparation of mono- and bis-NHC surface coordination. (b) FTIR spectra of SiO_2–700_, [Au(IMes)(Mes)], **1-Au**, and **2-Au**.

C, H, N, and Au elemental analysis of the resulting material **1-Au**, [(NHC)Au/SiO_2–700_] (see SI-1.5), is in agreement with the formation of a monografted species, with a surface density of ca. 0.4 Au nm^–2^. Furthermore, diffuse reflectance ultraviolet–visible (DRUV) spectroscopy of **1-Au** displays two bands at 255 and 270 nm, associated with ligand-to-metal charge transfer, consistent with previously reported molecular Au^I^ carbene complexes (Figure S2).^28^ Next, ^13^C solid-state NMR spectroscopy, recorded under ^13^C{^1^H} cross-polarization and magic angle spinning (CPMAS), of the corresponding ^13^C labeled surface sites 1*-Au (see SI for experimental details) displays one dominant peak at 169 ppm, attributed to the labeled NHC carbenic carbon bound to an Au(I) site (Figure 3a). A similar chemical shift is found in the corresponding molecular analogue [Au(IMes*)(OTBOS)] (164 ppm), having a tris(*tert*-butoxy) ligand in place of surface siloxy group (Figure 3d)^26^ and shifted by ca. 50 ppm with respect to the free carbene (219 ppm), consistent with binding on Au(I) surface sites.^29^

**Figure 3:**
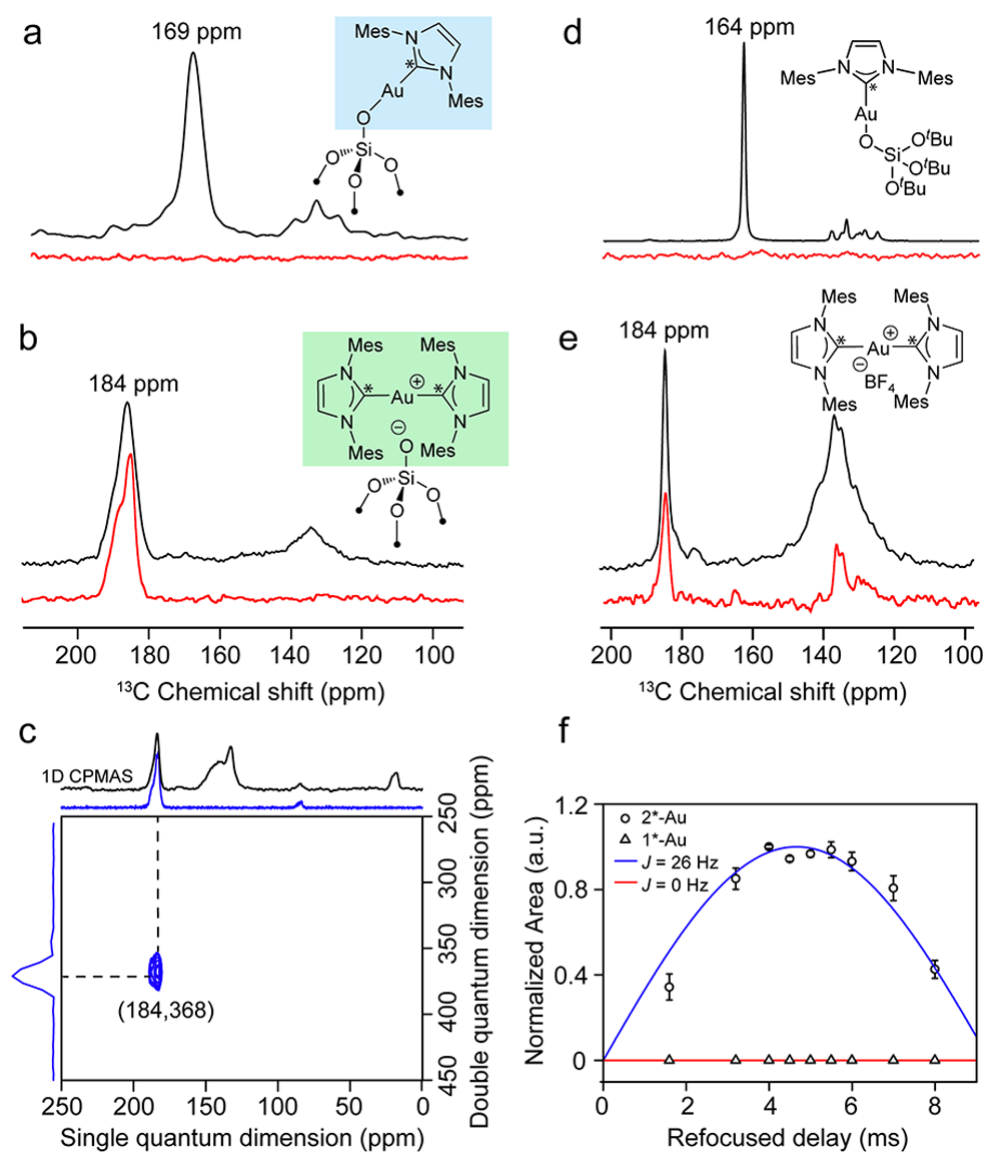
^13^C{^1^H} CPMAS (black) and ^13^C{^1^H} CP-INADEQUATE (red) spectra of (a) 1*-Au, (b) [Au(IMes*)(OTBOS)], (d) 2*-Au and (e) [Au(IMes*)_2_][BF_4_]. The spectra were collected at ∼298 K, MAS rates of 10 kHz, and CP contact time of 6 ms. (c) The 2D ^13^C{^1^H} CP-INADEQUATE spectrum (blue) and 1D ^13^C{^1^H} CPMAS spectrum (black) of 2*-Au. The spectra were collected at ∼110 K, MAS rates of 10 kHz. (f) The normalized area of the ^13^C NMR peak at 184 ppm as a function of refocused delay (τ) in the 1D ^13^C INADEQUATE experiment.

We next probed the reactivity of **1-Au** toward additional amounts of the IMes ligand. The resulting solid **2-Au**, [(IMes)_2_Au/SiO_2–700_], displays C, N, H, and Au elemental analysis (Au, 2.83; C, 7.48; N, 0.97; H, 0.71 wt %), consistent with the presence of two IMes per surface Au atom. In fact, the IR spectrum of **2-Au** displays an increased intensity of bands associated with organic ligands. Notably, the DRUV spectrum shows the same bands at 255 and 270 nm as in **1-Au**, but with different relative intensities, supporting the formation of different surface sites (Figure S3). Most notably, the ^13^C NMR spectrum of **1-Au** contacted with ^13^C labeled NHC shows that the peak at 169 ppm in **1-Au** is shifted to 184 ppm in **2-Au** (Figure 3b), and its intensity is greatly increased for the corresponding fully labeled compounds prepared from 1*-Au contacted with IMes*. We thus hypothesize that the reaction of the free carbene, IMes, with IMes–Au–OSi≡ could generate a Au(I) species stabilized by two NHC ligands, paralleling what is observed in surface science with metallic surfaces as well as an earlier report where bis-PMe_3_ Au(I) surface sites were observed upon contacting Au(I) surface sites with excess PMe_3_.^30^ Notably, the reaction of the molecular analogue [Au(IMes)(OTBOS)] with 1 equiv of IMes yields a similar NMR spectral signature with a peak at 184 ppm (Figure S4), as does [Au(IMes)_2_][BF_4_], supporting the formation of a bis-ligated cationic Au(I) species, Au(IMes)_2_^+^, along with a surface silicate as a counteranion (Figure 3e).

In order to confirm these assignments, we carried out ^13^C{^1^H} CP-INADEQUATE experiments,^31^ that enables us to distinguish between isolated and pairs of NHC ligands found in mono and bis NHC surface sites.^30^ Following calibration of this approach with molecular Au(I) complexes (see SI, Figures S5 and S8), we investigated 1*-Au vs 2*-Au: while the ^13^C 1D-INADEQUATE spectrum of 1*-Au shows no peak at 169 ppm consistent with the assignment to a monocoordinated NHC Au complex (Figure 3a, red), a peak at 184 ppm appears for 2*-Au (Figure 3b, red), confirming the formation of bis-NHC Au(I) species. In fact, the corresponding 2D-INADEQUATE spectrum of 2*-Au (Figure 3c) shows a cross-peak at 184 and 368 ppm in the single and double quantum frequency dimension respectively, unequivocally pointing to a spin–spin coupling between the two equivalent carbene carbons of 2*-Au. Further visualization of the difference between 1*-Au and 2*-Au is illustrated by tracking the integrated area of the peak at 184 ppm in the 1D-INADEQUATE spectrum as a function of increasing refocused delay (τ) (Figure 3f). While no change was seen for 1*-Au, it enables the estimation of the two-bond ^13^C scalar coupling constant in 2*-Au, ^2^*J*_(c-c)_ = 26 ± 3 Hz (see SI, Figures S6–S7). The relatively high value for ^2^*J*_(c-c)_ is in accordance with the directly bonded electronegative Au(I) cation.^32−34^

**Figure 4:**
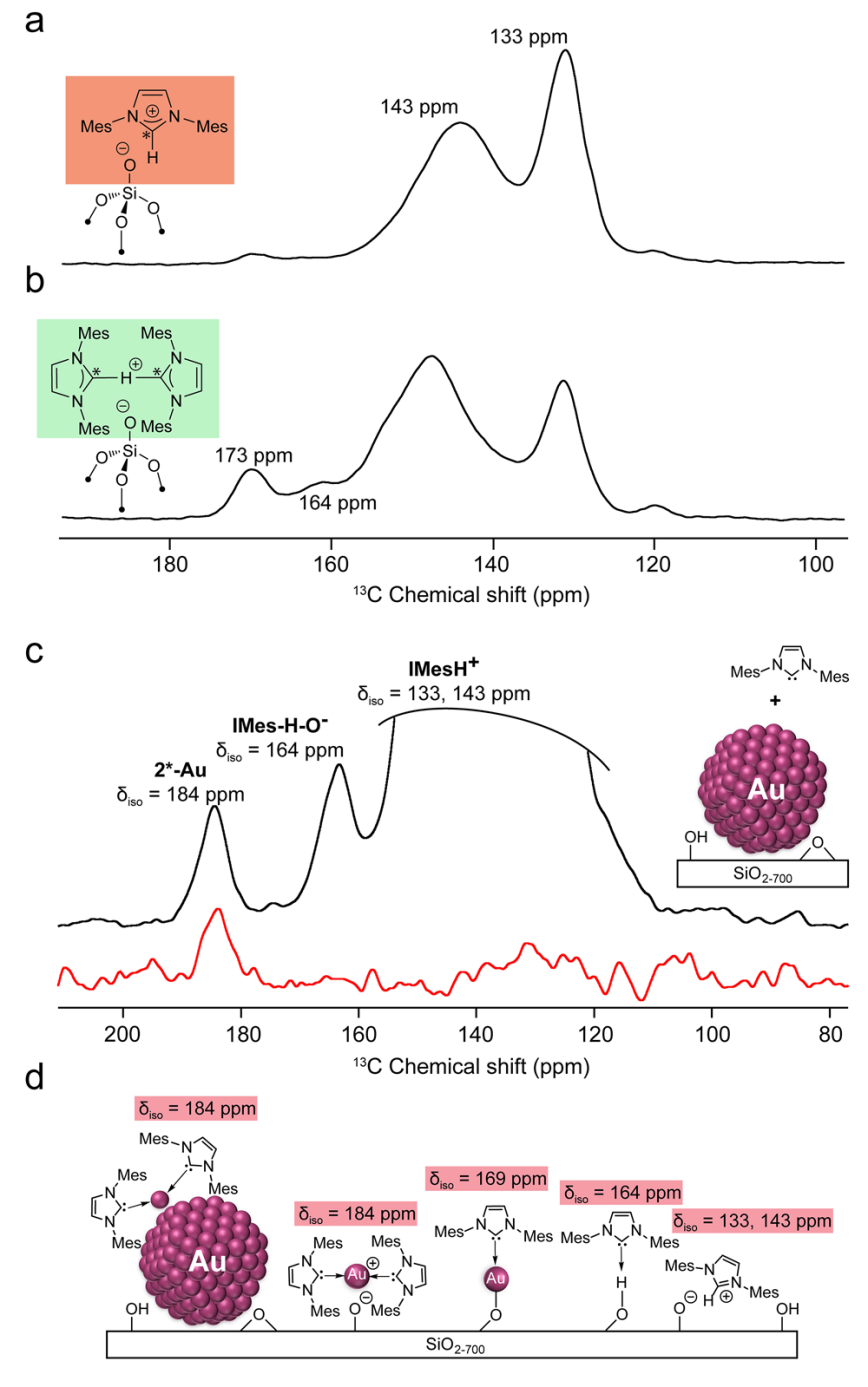
(a) ^13^C{^1^H} CPMAS spectrum of 1*-H and (b) 2*-H. (c) ^13^C{^1^H} CPMAS (black) and ^13^C-1D INADEQUATE (red) NMR spectra of IMes* functionalized AuNPs/SiO_2_. The spectra were collected at ∼110 K and MAS rates of 10 kHz. (d) Scheme depicting the various surface species that are present on the oxide surface.

Having established the formation of bis-NHC ligated cationic Au(I) surface sites for **2-Au**, we investigated the reactivity of the corresponding silica-supported Cu^I^ and Ag^I^ surface sites toward NHC. The reaction of IMes with **1-Cu** ([(IMes)Cu/SiO_2–700_]) and **1-Ag** ([(IMes)Ag/SiO_2–700_]), bearing ≡SiOCuIMes and ≡SiOAgIMes surface sites, afforded the corresponding solids **2-Cu** ([(IMes)_2_Cu/SiO_2–700_]) and **2-Ag** ([(IMes)_2_Ag/SiO_2–700_]), with bis-cationic surface sites according to extensive characterization (see SI, Figures S9–S14). Notably, the 2D-INADEQUATE spectrum taken for 2*-Ag (Figure S14b) is particularly illustrative, with a cross peak clearly indicating the formation of the bis-NHC Ag cationic species. Considering the isolobal analogy between coinage cations and protons, as recently shown for Ag,^37^ we also reinvestigated the reaction of IMes with surface silanols.^26^ Contacting SiO_2–700_ with IMes* to SiO_2_ (1 equiv. per surface silanol) yields a solid 1*-H (IMes*/SiO_2–700_), whose ^13^C{^1^H} CPMAS spectrum displays two peaks at 133 and 143 ppm, consistent with the formation of imidazolium surface sites, generated by deprotonation of the silanol by NHC^26^ (Figure 4a). However, the addition of one more equiv of IMes* to this mixture leads to the appearance of two new peaks at 164 and 173 ppm in the ^13^C{^1^H} CPMAS spectrum (Figure 4b). The minor peak at 164 ppm was correlated with IMes* H-bonded to surface silanols or to binding of IMes coordinated to Si(−O−)_4_ sites.^26^ Notably, the peak at 173 ppm was consistent with the formation of a bis-NHC proton complex. This assignment is fully in line with the pioneering report of Arduengo that isolated and fully characterized the corresponding molecular analog.^35^ The relatively low amount of this surface site is likely due to the competitive adsorption with imidazolium or a less energetically favorable situation.

Considering what is known about the different binding modes of NHC on metallic Au (111) from surface science studies, we also investigated the reaction of IMes* with silica-supported Au nanoparticles, AuNPs/SiO_2–700_ (2.0 ± 0.4 nm), prepared according to a previously reported procedure^27^ (see SI, Figures S15–S17). The ^13^C CPMAS spectrum (Figure 4c, black spectrum) of the resulting solid displays four peaks: three at 133, 143, and 164 ppm as found for IMes*/SiO_2_, along with a fourth peak at 184 ppm, which can be ascribed to the formation of bis-NHC Au(I) cationic species. This assignment is again further supported by the ^13^C 1D-INADEQUATE spectrum that displays a peak at 184 ppm (Figure 4c, red spectrum).

The observation of this bis-NHC cationic Au(I) species is noteworthy and raises questions regarding its location, i.e., whether it is adsorbed at a negatively charged NP counterpart or at surface silicates. Considering that Au is mostly in the form of Au(0) in AuNPs/SiO_2_^27^, this significant amount of bis-NHC cationic Au(I) species likely originates from etching of the AuNPs surface^36^ in conjunction with migration of this species to the interface, possibly via exchange with a surface hydroxyl groups of silica (Figure 4d). While difficult to assess experimentally, this last result already indicates that bis-ligated surface sites are particularly stable when coinage metals or protons are at play, independent of the type of surfaces.

In conclusion, this work has identified the NMR spectroscopic signatures of the mono- and bis-NHC Au(I) species on supported systems, thanks to the use of state-of-the-art solid-state NMR spectroscopy, benchmarked on ^13^C labeled molecular models. The ease of formation of cationic bis-NHC ligated motifs was demonstrated across group 11 and also occurred for surface protons. The ^13^C NMR signature of the bis-NHC Au motif was also found as the pertinent signature of NHC coated AuNPs supported on SiO_2_. These observations enable us to bridge the gap between well-defined metal surfaces to NPs and surface sites, showcasing that the bis NHCs coordination on metal sites can readily be achieved across ranges of systems, from well-defined facets to metal sites in NPs and dispersed on oxide supports like silica. These results showcase that one must consider such motifs when metal nanoparticles are in contact with NHC ligands, given the propensity of forming such bis-NHC cationic species as already discussed on metallic surfaces, further demonstrating the rich coordination chemistry of NHC ligands. We are currently exploring the generality of these phenomena with other metals.

## Supporting Information Available

Additional experimental details, materials, and methods, including spectral data, TEM images and crystal structures. (PDF)
