# Supplementary material for: NMR Spectroscopic Signatures of Cationic Surface Sites from Supported Coinage Metals Interacting with N-Heterocyclic Carbenes
Source: J Am Chem Soc. 2024 Mar 1;146(10):6466–70. doi: 10.1021/jacs.4c00200 (PMC10941179; doi:10.1021/jacs.4c00200)
Supplement: Supplementary file 1 — ja4c00200_si_001.pdf [file ja4c00200_si_001.pdf]

NMR Spectroscopic Signatures of Cationic Surface Sites from Supported Coinage Metals Interacting with N-Heterocyclic Carbenes

Shahar Dery[a], Weicheng Cao[a], Chengbo Yao[a], and Christophe Copéret[a]*

[a]*Department of Chemistry and Applied Biosciences, ETH Zürich, CH-8093 Zürich, Switzerland*

Supporting information

Table of Content

1. Experimental Procedures

1.1 Materials …………………………………………………………………………… 3

1.2 General methods ……………………………………………………………………… 4

1.3 Solid-state NMR spectroscopy experiments ………………………………………... 5

1.4 Estimation of J coupling constant by 1D-INADEQUATE experiments …………… 5

1.5 Synthetic procedures…………………..………………………………………………..6

2. XRD studies

2.1 Crystal structures……………………………………………………………………21

2.2 XRD reports………………………………………………………………………… 22

3. References……………………………………………………………………………… 24

1. Experimental Procedures

1.1. Materials

Unless otherwise noted, all experiments were conducted with dry, oxygen-free solvents using standard Schlenk techniques or in Ar-filled gloveboxes (MBraun UNIlab). Toluene and pentane were purified by passage through a double solvent purification system (MBraun MB-SPS-800) on alumina columns (MBraun SPS alumina columns) under N_2 and freeze-pump thaw degassed before utilization. THF was distilled over Ar from purple Na/benzophenone and stored over 3 Å sieves. Benzene-d6 was vacuum-distilled from purple Na/benzophenone. Dry deuterated Chloroform ($CDCl_3$) was obtained via vacuum distillation from CaH_2. Acetone (C_3H_6O) was dried by stirring it with activated 3 and 4 Å molecular sieves for a week. All solvents were further degassed via three freeze-pump-thaw cycles and stored over 4 Å molecular sieves after being transferred to a glove box. Reagents were obtained from commercial suppliers and used as received. Synthetic procedures for non-labeled, respectively C_2 ^{13}C-labeled, 1,3-bis(2,4,6-trimethylphenyl)imidazolium chloride (IMesHCl, resp. IMes*HCl) and 1,3-bis(2,4,6-trimethylphenyl)imidazol-2-ylidene (IMes, resp. IMes*) were adapted from procedures of the literature[1]. Silica (Aerosil Degussa, 200 m^2g^{-1}) was compacted with deionized water, dried at 100 °C for 7 days, crushed, and sieved (250−400 µm). Quantification of the –OH density of the oxide supports was performed through the reaction of $[Mg(CH_2Ph)_2(THF)_2]$ with a known amount of the oxide. The amount of toluene liberated was quantified by NMR in C_6D_6 using ferrocene as an internal standard. For this purpose, a recycle delay (D_1) of 59 seconds was used. Gold mesityl (**[Au$_5$Mes$_5$)]**), **[Au(IMes)Cl]**, **[Au(IMes*)Cl]**, **[Au(IMes*)$_2$][BF$_4$]** prepared following reported procedures[2-4] using non-labeled IMesHCl, ^{13}C-labeled IMes*HCl, imidazolium salt, respectively. Partially dehydroxylated silica (**SiO$_{2-700}$**, 0.35 mmolOH·g^{-1}) and silica-supported gold nanoparticles (AuNPs/SiO_2) were prepared as previously reported[2]. Silver and copper complex **[Ag(IMes*)Cl]**, **[Ag(IMes*)(Mes)]**, **[Ag(IMes)$_2$][OTf]**, **[Cu(IMes*)Cl]**, **[Cu(IMes*)(Mes)]**, **[Cu(IMes)$_2$][OTf]** were synthesized according to previous reports[5-7].

1.2. General Methods

Solution NMR spectra were recorded at room temperature (298 K) on a Bruker 300 or 500 MHz solution state spectrometer equipped with a broadband probe or a broadband cryoprobe (^{13}C, ^{1}H). ^{1}H and ^{13}C chemical shifts are referenced relative to residual solvent peaks[8]. Chemical shifts are reported in parts per million (ppm). Where appropriate, signal multiplicity has been condensed to a single letter format, i.e.: s=singlet, d=doublet, t=triplet, q=quartet, m=multiplet. Solvent signals are denoted accordingly. Unless otherwise specified, ^{13}C spectra were recorded using 2048 scans, and ^{1}H spectra were acquired using 64 scans. The Small Molecule Crystallography Center (SMoCC) of ETH Zürich provided the equipment for single crystal structure determination. XRD data was recorded on a Rigaku XtaLAB Synergy-S single-crystal diffractometer. Suitable crystals were selected and tipmounted on a MiTeGen Pin covered with Paratone Oil. Using Olex2[9], the structure was solved with the SHELXT[10] structure solution program and refined with the SHELXL[11] refinement package using CGLS minimization. The hydrogen atoms were placed in ideal positions and refined as riding atoms. Elemental Analysis was provided by the in-house Molecular and Biomolecular Analysis Service (MoBiAS) of ETH Zürich and Analytisches Labor Pascher in Remagen, Germany. Infrared (IR) spectra of the molecular compounds were collected under an inert atmosphere using a Bruker Alpha spectrometer in attenuated total reflectance (ATR) mode, equipped with a diamond ATR module (deuterated triglycine sulfate (DTGS) detector, 2 cm^{-1} spectral resolution, 4000-400 cm^{-1}, average of 64 scans). Transmission-IR were recorded on Bruker FT-IR Alpha spectrometer equipped with RockSolid interferometer, DTGS (deuterated triglycine sulfate) detector, SiC globar source; solid samples were mounted on a magnetic pellet holder. A typical experiment consisted of the measurement of transmission in 64 scans in the region from 4000 to 400 cm^{-1}. Spectra were analyzed using OPUS 8 Software. Spectra of silica-supported samples are normalized to the Si-O-Si overtone peak maximum at 1868 cm^{-1}. Diffuse reflectance UV-vis (DRUV) spectra were recorded on an Agilent Cary 5000 UV-Vis-NIR spectrometer equipped with a Praying MantisTM Diffuse Reflection accessory in absolute %R under double beam mode, at a scan rate of 600 $nm\cdot min^{-1}$ and a resolution of 1 nm. The samples were prepared in a glovebox by grinding the material in an agate mortar. The obtained mixture was layered in an ambient chamber for Praying MantisTM (Harrick), which was sealed under the inert atmosphere of the glovebox. The source changeover was performed at 350 nm and the detector/grating changeover was performed at 860 nm. Reflectance (R) data were converted to Kubelka-Munk (KM) units taking pure PTFE powder as the reference reflector. Transmission electron microscope (TEM) images were recorded on a FEI Talos

F200X instrument operated at 200 keV. Powdered samples were mixed in solid form with a Lacey-C 400 mesh Cu grid inside of a glovebox under an atmosphere of Ar before mounted on a vacuum transfer tomography holder from Fischione Instruments (model #2560) inside the glove box which was subsequently transferred to the chamber of the TEM in the absence of air. All given values for size distributions assume a normal distribution. The nanoparticle diameter determination to obtain the particle size distribution (PSD) was done by manual measurement with the standard software ImageJ (version 1.52a). The "±" in the depicted particle size distributions indicates the standard deviation of the mean.

1.3. Solid-state NMR experiments

All the solid-state NMR experiments were performed on a Bruker 9.4 T NMR spectrometer, using a 3.2 mm low-temperature MAS probe. For the $^{13}C\{^{1}H\}$ cross polarization magic angle spinning (CPMAS) experiments at room temperature (≈298 K), the MAS rate was 16 kHz, the CP contact time was varied from 2-5 ms as optimized for different samples and the ^{1}H decoupling power was 120 kHz. For $^{13}C\{^{1}H\}$ CPMAS and INADEQUATE experiments at low temperature (≈110 K), the MAS rate was 10 kHz, the CP contact time was 6 ms and the ^{1}H decoupling power was 65 kHz. The ^{13}C chemical shifts were calibrated to the methylene signal of adamantane at 38.5 ppm. The refocused version of CP-INADEQUATE pulse sequence[12] was used in this work. The 1D-INADEQUATE spectra with different refocused delays were collected, respectively. The optimized refocused delay was used for the further 2D-INADEQUATE experiments. All the samples for NMR measurements were prepared in an argon-filled glove box.

1.4 Estimation of J coupling constant by 1D-INADEQUATE experiments

In INADEQUATE experiments, the refocused delay in a spin echo is usually set equal to 1/4J for the creation of DQ coherence, where J is the average spin-spin coupling constant. By analyzing the dependence of signal intensity on the refocused delay in 1D INADEAUQTE, the average coupling constant J can be possibly estimated. In addition, it was shown that the dependence on the refocused delay in the spin echo can be complicated by the chemical shift difference, and expressed as followed[13, 14]:

$$I = 2cos^2 2\theta \sin[J\tau] - sin2\theta(1 - sin2\theta)\sin[(C - J)\tau] - sin2\theta(1 + sin2\theta)\sin[(C - J)\tau]$$

where J is the coupling constant, $\tan 2\theta = J/\Delta$, $C = \sqrt{\Delta^2 + J^2}$, and Δ is the chemical shift difference. By aligning the experimental results of the 1D-INADEQUATE, one can deduce the corresponding J coupling constant. The results are shown in Figure S6-S7.

1.5 Synthetic procedures

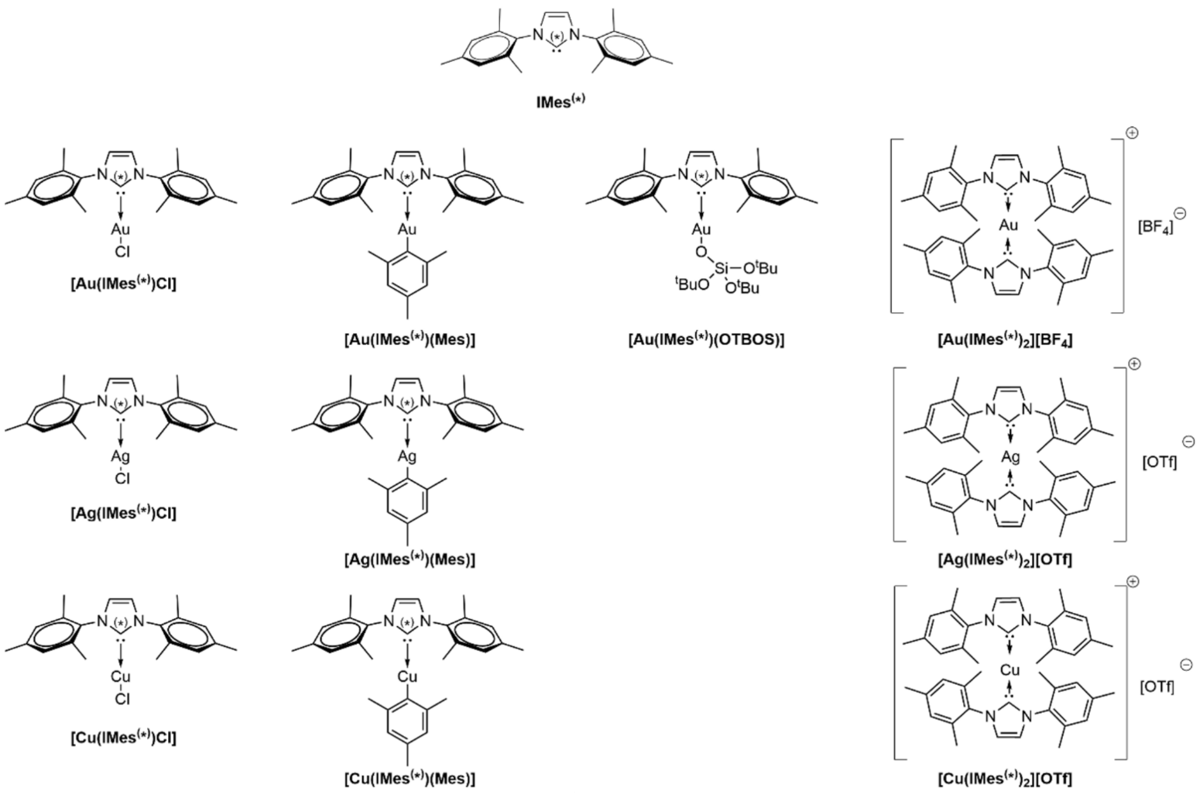

Figure S1. The studied molecular compounds.

[Au(IMes)(Mes)]

To an amber vial was added IMesAuCl (100 mg, 0.186 mmol) followed by THF (ca. 5 mL). To the suspension was added dropwise a solution of $Mes_2Mg(THF)_2$ (77.8 mg, 0.186 mmol) in THF (ca. 3 mL). The mixture was stirred for 20 h before 2 drops of dioxane were added to precipitate $[MgCl_2(C_4H_8O_2)]_n$. After filtering the reaction mixture through Celite® solvent was removed under reduced pressure to yield a white product. The crude product was dissolved in toluene (5 mL) and cooled to -35 ^{o}C for 24 hours. Colorless cubic crystals were obtained in a yield of 62% (73 mg, 0.115 mmol). XRD quality crystals were obtained from recrystallization in toluene.

^{1}*H-NMR* (25 °C, 300 MHz, C_6D_6): δ 7.00 (s, 2H, Au-Mesityl), δ 6.75 (s, 4H, NHC-Mes), δ 6.05 (s, 2H, backbone C_2H_2), δ 2.41 (s, 6H, Ar-CH_3 (4–position) (NHC)), δ 2.28 (s, 3H, Ar-CH_3 (4–position) Au-Mesityl)), δ 2.10 (s, 6H, Ar-CH_3 (2,6-position) (Au-Mesityl)), δ 2.04 (s, 12H, Ar-CH_3 (2,6-position) (NHC)).
^{13}*C-NMR* (25 °C, 75 MHz, C_6D_6): 199.4 (s, C carbene), 167.7 (s, *ipso* mesityl *C*), 146.3, 139.0, 135.8, 135.0, 132.8, 131.4, 129.1, 126, 120.9, 26.4, 21.4, 20.9, 17.7.
IR (ATR-IR) [cm^{-1}] 3160, 3113 (m, alkene C-H str.), 3060 – 2800 (m, C-H str.), 2726 (w, Mes C-H str.), 1610 (w, C=C str.), 1484 (w, arom. C-C str.), 1402 (s, C-H bend.), 1232 (s, C-N str.), 982/848/739 (s, alkene C-H bend.).
Elemental Analysis C: 58.51% H: 5.33% N: 4.70% (Calc.: 58.06%/ 5.68%/ 4.51%).

[Au(IMes*)(Mes)]
The complex was prepared following the same procedure using the ^{13}C-labeled IMes* ligand instead of IMes.
^{1}*H-NMR* (25 °C, 300 MHz, C_6D_6): δ 7.00 (s, 2H, Au-Mesityl), δ 6.75 (s, 4H, NHC-Mes), δ 6.06 (d, 2H, *J* = 2.2 Hz, backbone C_2H_2), δ 2.41 (s, 6H, Ar-CH_3 (4–position) (NHC)), δ 2.28 (s, 3H, Ar-CH_3 (4–position) Au-Mesityl)), δ 2.10 (s, 6H, Ar-CH_3 (2,6-position) (Au-Mesityl)), δ 2.04 (s, 12H, Ar-CH_3 (2,6-position) (NHC)).
^{13}*C-NMR* (25 °C, 75 MHz, C_6D_6): 199.4 (s, C carbene), 167.7 (s, *ipso* mesityl *C*), 146.3, 139.0, 135.8, 135.0, 132.8, 131.4, 129.1, 126, 120.9, 26.4, 21.4, 20.9, 17.7.
Elemental Analysis C: 58.31% H: 5.85% N: 4.78% (Calc.: 58.11%/ 5.63%/ 4.50%).

[Au(IMes)(OTBOS)]
To an amber vial was added IMesAuCl (100 mg, 0.186 mmol) followed by THF (ca. 5 mL). To the suspension was added NaO^{t}Bu (21.5 mg, 0.22 mmol) in THF (ca. 3 mL). The mixture was stirred for 2 h. After filtering the reaction mixture through Celite® the solution was taken to dryness, leaving a pale-orange solid. The crude product was dissolved in toluene, filtered, dried, and finally washed with pentane to obtain a yield of 70% (57 mg, 0.12 mmol). XRD quality crystals were obtained from recrystallization in toluene.
^{1}*H-NMR* (25 °C, 300 MHz, C_6D_6): δ 6.67 (s, 4H, NHC-Mes), δ 5.96 (backbone C_2H_2), δ 2.08 (s, 6H, Ar-CH_3 (4–position) (NHC)), δ 1.92 (s, 12H, Ar-CH_3 (2,6-position) (NHC)), δ 1.49 (s, 27H, -CH_3).

^{13}C-NMR (25 °C, 300 MHz, C_6D_6): 169.60 (s, C carbene), 138.94, 135.17 (backbone C_2H_2), 134.44, 129.24, 120.82, 70.31, 31.93, 20.69, 17.35.

IR (ATR-IR) [cm^{-1}] 3175/3130 (w, alkene C-H str.), 3000-2800 (m, C-H str.), 2733 (w, Mes C-H str.) 1487 (m, arom. C-C str.), 1357 (m, C-H bend.), 1233/1192 (m, C-O str.), 1001 (s, Si-O-C str.), 815/692 (m, Si-O str.)

Elemental Analysis C: 52.01% H: 7.04% N: 3.75% (Calc.: 51.82% / 6.72% / 3.66%).

[Au(IMes*)(OTBOS)]

The complex was prepared following the same procedure using the ^{13}C-labeled IMes* ligand instead of IMes.

^{1}H-NMR (25 °C, 300 MHz, C_6D_6): δ 6.67 (s, 4H, NHC-Mes), δ 5.96 (d, 2H, *J* = 2.2 Hz, backbone C_2H_2), δ 2.08 (s, 6H, Ar-CH_3 (4–position) (NHC)), δ 1.92 (s, 12H, Ar-CH_3 (2,6-position) (NHC)), δ 1.49 (s, 27H, -CH_3).

^{13}C-NMR (25 °C, 300 MHz, C_6D_6): 169.60 (s, C carbene), 138.94, 135.17 (d, *J* = 9 Hz, backbone C_2H_2), 134.44, 129.24, 120.82, 70.31, 31.93, 20.69, 17.35.

Elemental Analysis C: 52.15 % H: 6.91% N: 3.87% (Calc.: 51.88% / 6.66% / 3.66%).

1-Au = [(IMes)Au]/SiO_2

[Au(IMes)Mes] (65.2 mg, 0.11 mmol) is dissolved in 3 mL benzene. SiO_{2-700} (280 mg, 0.1 mmolOH) are added to this solution. The mixture is stirred at 120 rpm at room temperature for 6 hours. The solid is then allowed to decant and the supernatant pipetted out. The solid is then washed intermittently 3 times with 5 mL benzene and 5 mL of pentane, dried under vacuum, then high vacuum to afford a white powder.

Elemental Analysis found: C, 3.82; H, 0.40; N, 0.48; Au, 2.80 (wt%), (Calc. for $C_{21}H_{24}N_2Au$: C, 50.30; H, 4.82; N, 5.59; Au, 39.28).

1*-Au = [(IMes*)Au]/SiO_{2-700} was prepared following the same procedure using the ^{13}C-labeled IMes* ligand instead of IMes.

Elemental Analysis found: C, 3.90; H, 0.46; N, 0.45; Au, 2.82 (wt%), (Calc. for $C_{21}H_{24}N_2Au$: C, 50.38; H, 4.77; N, 5.57; Au, 39.23).

2-Au = [$(IMes)_2$Au]/SiO_{2-700}

IMes (30 mg, 0.10 mmol) is dissolved in 3 mL benzene. **1-Au** (280 mg) are added to this solution. The mixture is stirred at 120 rpm at room temperature for 3 hours. The solid is then allowed to decant and the supernatant pipetted out. The solid is then washed intermittently 3 times with 5 mL benzene and 5 mL of pentane, dried under vacuum, then high vacuum to afford a white powder.

Elemental Analysis found: C, 7.48; H, 0.71; N, 0.97; Au, 2.83 (wt%), (Calc. for $C_{42}H_{48}N_4Au$: C, 62.79; H, 5.95; N, 7.95; Au, 24.44).

2*-Au = [(IMes*)$_2$Au]/SiO$_{2-700}$ was prepared following the same procedure using the ^{13}C-labeled IMes* ligand instead of IMes or **1*-Au** instead of **1-Au**.

Elemental Analysis found: C, 7.24; H, 0.67; N, 0.80; Au, 2.85 (wt%), (Calc. for $C_{42}H_{48}N_4Au$: C, 62.64; H, 5.94; N, 7.92; Au, 24.38).

1-Ag = [(IMes)Ag]/SiO$_2$

The procedure was adapted from our previous work[5]. [Ag(IMes)Mes] (58.3 mg, 0.11 mmol) is dissolved in 3 mL benzene. SiO_{2-700} (280 mg, 0.1 mmolOH) are added to this solution. The mixture is stirred at 120 rpm at room temperature for 3 hours. The solid is then allowed to decant and the supernatant pipetted out. The solid is then washed intermittently 3 times with 5 mL benzene and 5 mL of pentane, dried under vacuum, then high vacuum to afford a white powder.

Elemental Analysis found: C, 6.50; H, 0.64; N, 0.85; Ag, 2.7 (wt%), (Calc. for $C_{21}H_{24}N_2Ag$: C, 61.18; H, 5.87; N, 6.79; Ag, 26.16).

1*-Ag = [(IMes*)Ag]/SiO$_{2-700}$ was prepared following the same procedure using the ^{13}C-labeled IMes* ligand instead of IMes.

Elemental Analysis found: C, 6.87; H, 0.68; N, 0.89; Ag, 2.74 (wt%), (Calc. for $C_{21}H_{24}N_2Ag$: C, 61.39; H, 5.82; N, 6.78; Ag, 26.13).

2-Ag = [(IMes)$_2$Ag]/SiO$_{2-700}$

IMes (30 mg, 0.10 mmol) is dissolved in 3 mL benzene. **1-Ag** (280 mg) are added to this solution. The mixture is stirred at 120 rpm at room temperature for 3 hours. The solid is then allowed to decant and the supernatant pipetted out. The solid is then washed intermittently 3 times with 5 mL benzene and 5 mL of pentane, dried under vacuum, then high vacuum to afford a white powder.

Elemental Analysis found: C, 13.62; H, 1.46; N, 1.41; Ag, 2.89 (wt%), (Calc. for $C_{42}H_{48}N_4Ag$: C, 70.38; H, 6.75; N, 7.82; Ag, 15.05).

2*-Ag = [(IMes*)$_2$Ag]/SiO$_{2-700}$ was prepared following the same procedure using the ^{13}C-labeled IMes* ligand instead of IMes or **1*-Ag** instead of **1-Ag**.
Elemental Analysis found: C, 13.98; H, 1.48; N, 1.60; Ag, 2.93 (wt%), (Calc. for $C_{42}H_{48}N_4Ag$: C, 70.54; H, 6.69; N, 7.81; Ag, 15.02).

1-Cu = [(IMes)Cu]/SiO$_2$
The procedure was adapted from our previous work[6]. [Cu(IMes)Mes] (53.5 mg, 0.11 mmol) is dissolved in 3 mL benzene. SiO_{2-700} (280 mg, 0.1 mmolOH) are added to this solution. The mixture is stirred at 120 rpm at room temperature for 3 hours. The solid is then allowed to decant and the supernatant pipetted out. The solid is then washed intermittently 3 times with 5 mL benzene and 5 mL of pentane, dried under vacuum, then high vacuum to afford a white powder.
Elemental Analysis found: C, 6.48; H, 0.60; N, 0.79; Cu, 1.57 (wt%), (Calc. for $C_{21}H_{24}N_2Cu$: C, 68.54; H, 6.57; N, 7.61; Cu, 17.27).

1*-Cu = [(IMes*)Cu]/SiO$_{2-700}$ was prepared following the same procedure using the ^{13}C-labeled IMes* ligand instead of IMes.
Elemental Analysis found: C, 7.42; H, 0.70; N, 0.93; Cu, 1.62 (wt%), (Calc. for $C_{21}H_{24}N_2Cu$: C, 68.72; H, 6.51; N, 7.60; Cu, 17.26).

2-Cu = [(IMes)$_2$Cu]/SiO$_{2-700}$
IMes (30 mg, 0.10 mmol) is dissolved in 3 mL benzene. **1-Cu** (280 mg) are added to this solution. The mixture is stirred at 120 rpm at room temperature for 3 hours. The solid is then allowed to decant and the supernatant pipetted out. The solid is then washed intermittently 3 times with 5 mL benzene and 5 mL of pentane, dried under vacuum, then high vacuum to afford a white powder.
Elemental Analysis found: C, 12.86; H, 1.33; N, 1.48; Cu, 1.6 (wt%), (Calc. for $C_{42}H_{48}N_4Cu$: C, 75.07; H, 7.20; N, 8.33; Cu, 9.45).

2*-Cu = [(IMes*)$_2$Cu]/SiO$_{2-700}$ was prepared following the same procedure using the ^{13}C-labeled IMes* ligand instead of IMes or 1*-Cu instead of 1-Cu.

Elemental Analysis found: C, 12.98; H, 1.26; N, 1.42; Cu, 1.67 (wt%), (Calc. for $C_{42}H_{48}N_4Cu$: C, 75.02; H, 7.16; N, 8.30; Cu, 9.43).

1*-H = IMes*/SiO_{2-700}

To a solution of IMes* (30 mg; 0.1 mmol) in 5 mL benzene is added SiO_{2-700} (280 mg). The mixture is stirred at 120 rpm at room temperature for 6h. The solid is then allowed to decant and the supernatant pipetted out. The solid is then washed intermittently 3 times with 5 mL benzene and 5 mL of pentane, dried under vacuum, then high vacuum to afford a white powder.
Elemental Analysis found: C, 5.69; H, 0.53; N, 0.59 (wt%), (Calc. for $C_{21}H_{24}N_2$: C, 82.50; H, 7.95; N, 9.20).

2*-H = [(IMes*)$_2$H]/SiO_{2-700}

IMes (30 mg, 0.10 mmol) is dissolved in 3 mL benzene. 1-Cu (280 mg) are added to this solution. The mixture is stirred at 120 rpm at room temperature for 3 hours. The solid is then allowed to decant and the supernatant pipetted out. The solid is then washed intermittently 3 times with 5 mL benzene and 5 mL of pentane, dried under vacuum, then high vacuum to afford a white powder.
Elemental Analysis found: C, 10.57; H, 1.21; N, 1.34 (wt%), (Calc. for $C_{21}H_{24}N_2$: C, 82.50; H, 7.95; N, 9.20).

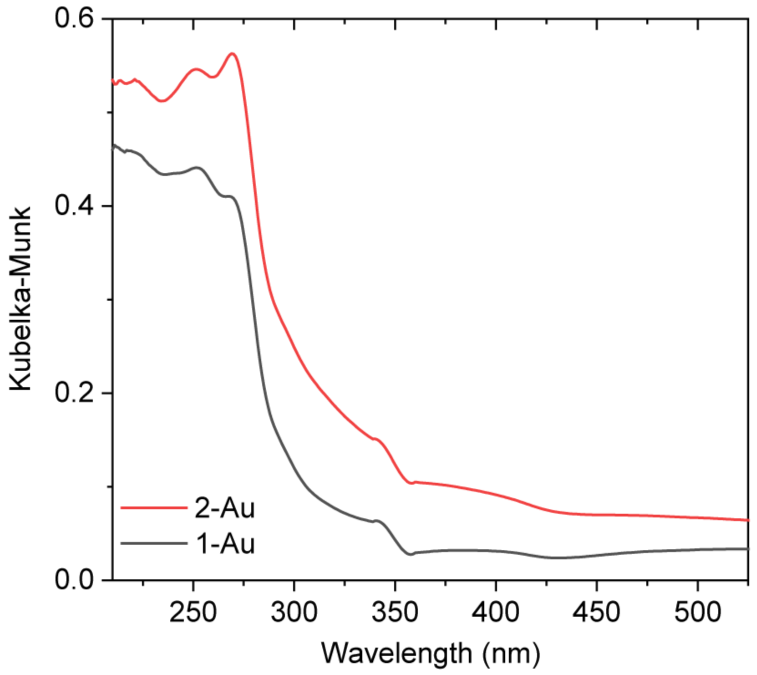

Figure S2. DRUV spectra of **1-Au** and **2-Au**.

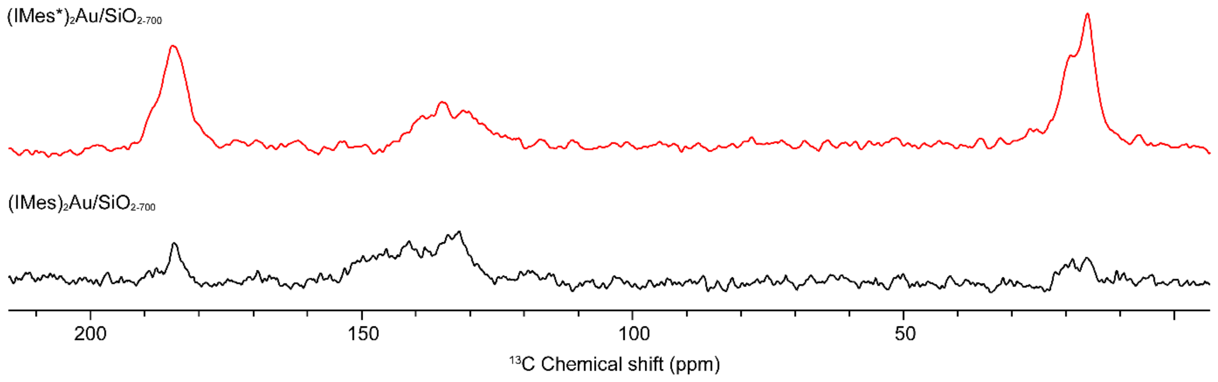

Figure S3. $^{13}C\{^{1}H\}$ CPMAS spectrum of **2-Au** (black) **2*-Au** (red). The spectra were collected at ~298 K, MAS rates of 10 kHz, and CP contact time of 2 ms.

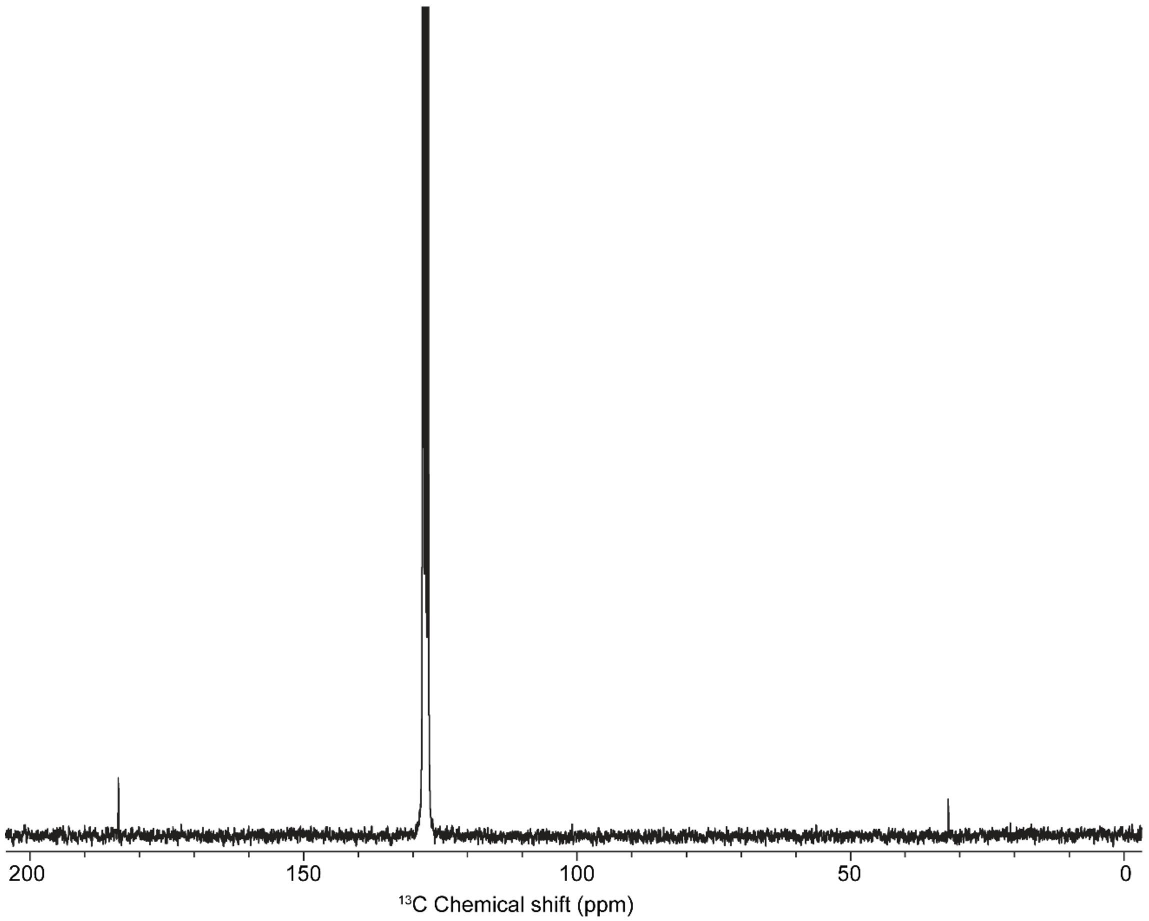

Figure S4. Solution state ^{13}C NMR spectrum of the product resulting from reaction of 1 equiv. [Au(IMes)(OTBOS)] and 1 equiv. of IMes*. A peak at 184 ppm suggests the formation of the bis-NHC Au species.

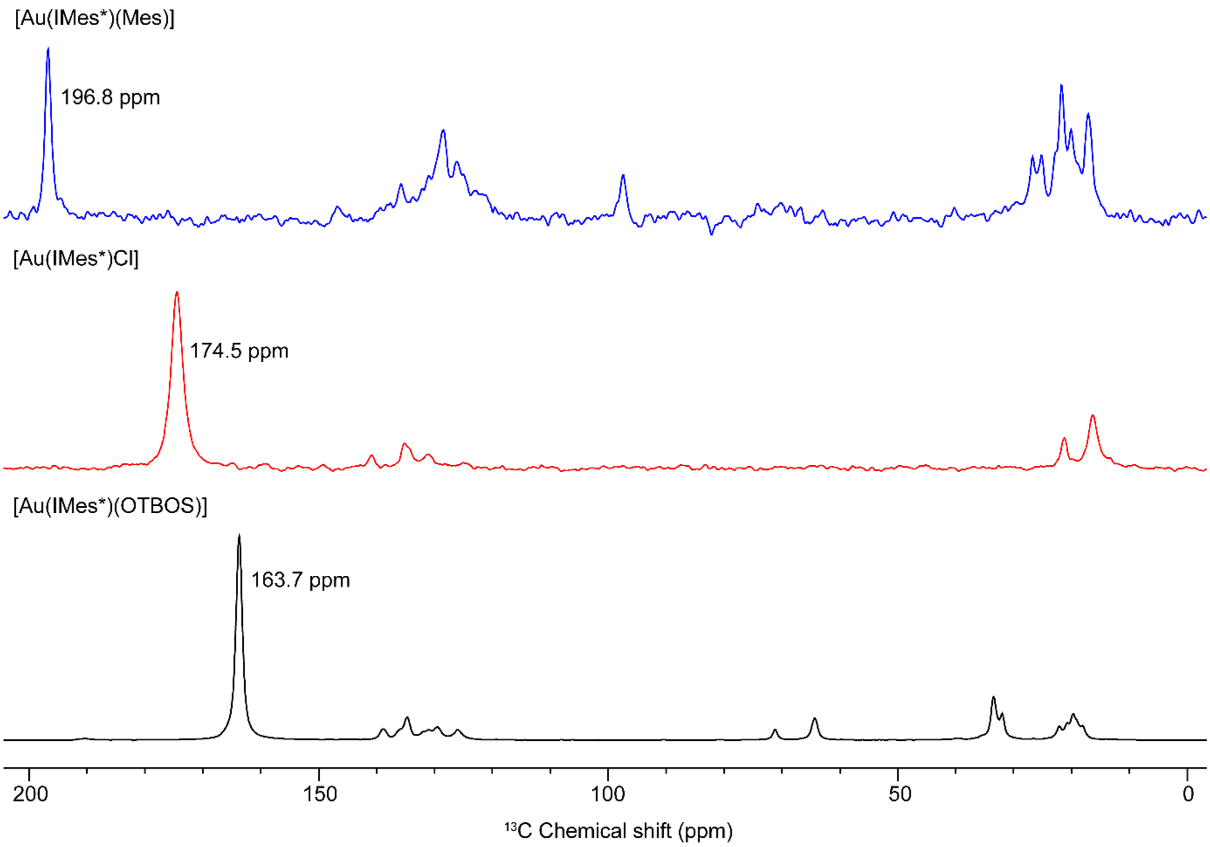

Figure S5. $^{13}C\{^{1}H\}$ CPMAS spectrum of [Au(IMes*)(OTBOS)] (black), [Au(IMes*)Cl] (red), [Au(IMes*)(Mes)] (blue). The spectra were collected at ~298 K, MAS rates of 18 kHz, and CP contact time of 2 ms.

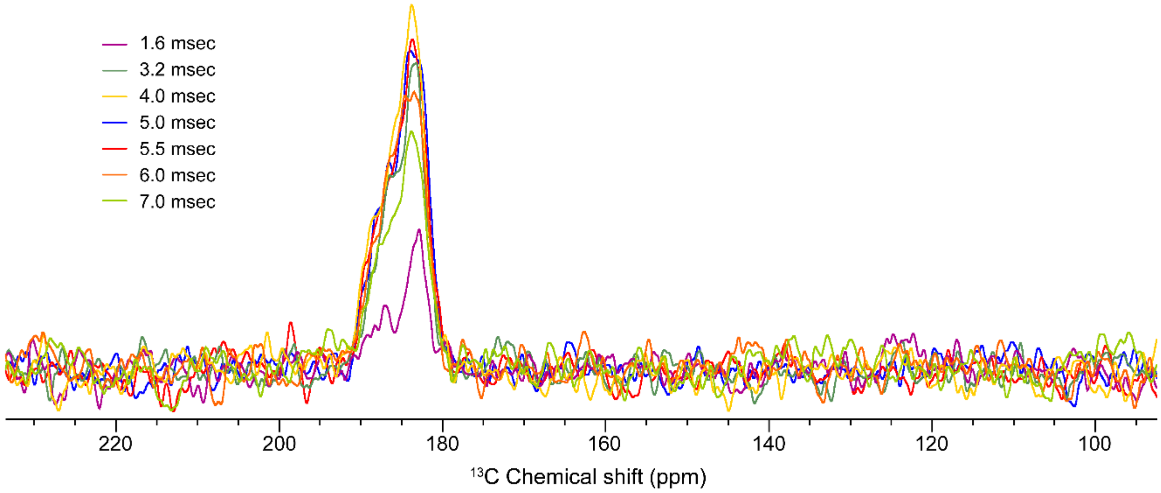

Figure S6. 1D ^{13}C INADEQUATE spectra of 2*-Au collected under different refocused delay times (see legend). The spectra were collected at ~110 K and MAS rates of 10 kHz.

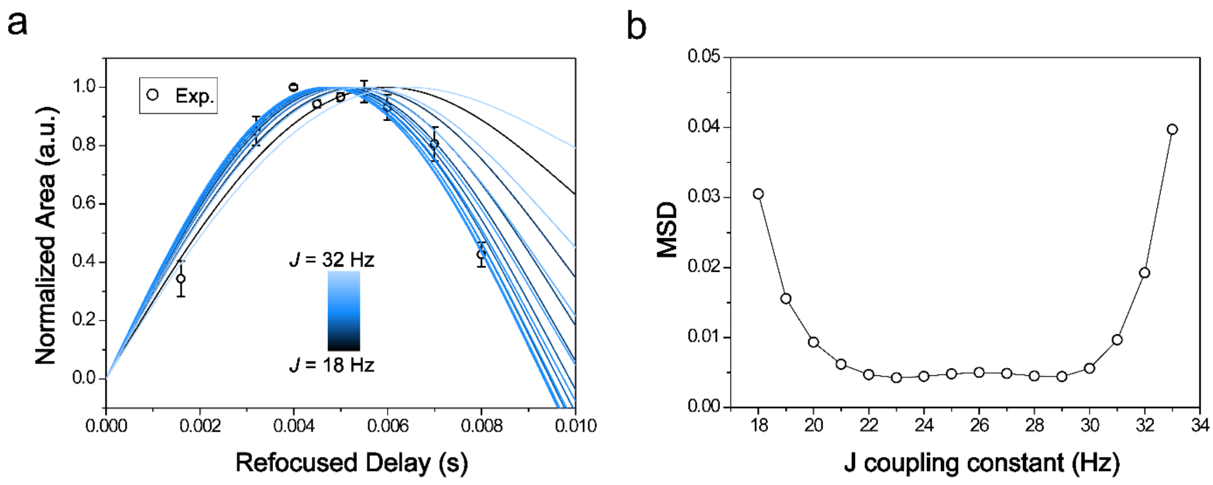

Figure S7. (a) The normalized integrated area of the peak at 184 ppm in the 1D-INADEQUATE spectrum of **2*-Au** with various refocused delays (circle). The calculated results corresponding to different J-coupling constants (18 – 32 Hz) with Δ=310 Hz were indicated by lines. (b) The mean square deviation (MSD) between calculated and experimental results shown in (a) indicating the estimated ^{13}C-^{13}C J coupling constant in **2*-Au** is 26 ± 3 Hz.

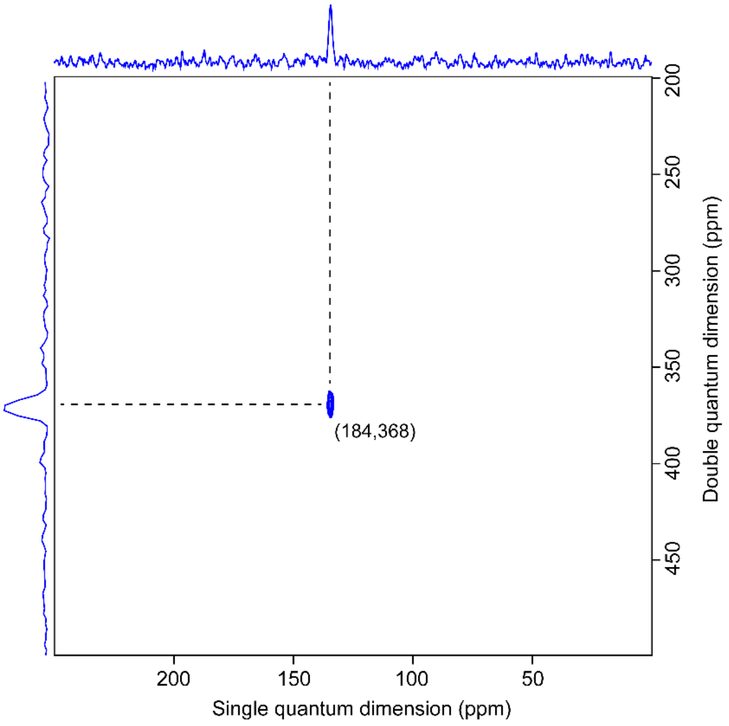

Figure S8. 2D ^{13}C INADEQUATE spectrum of [Au(IMes*)$_2$][BF$_4$], The spectra were collected at ~110 K and MAS rates of 10 kHz.

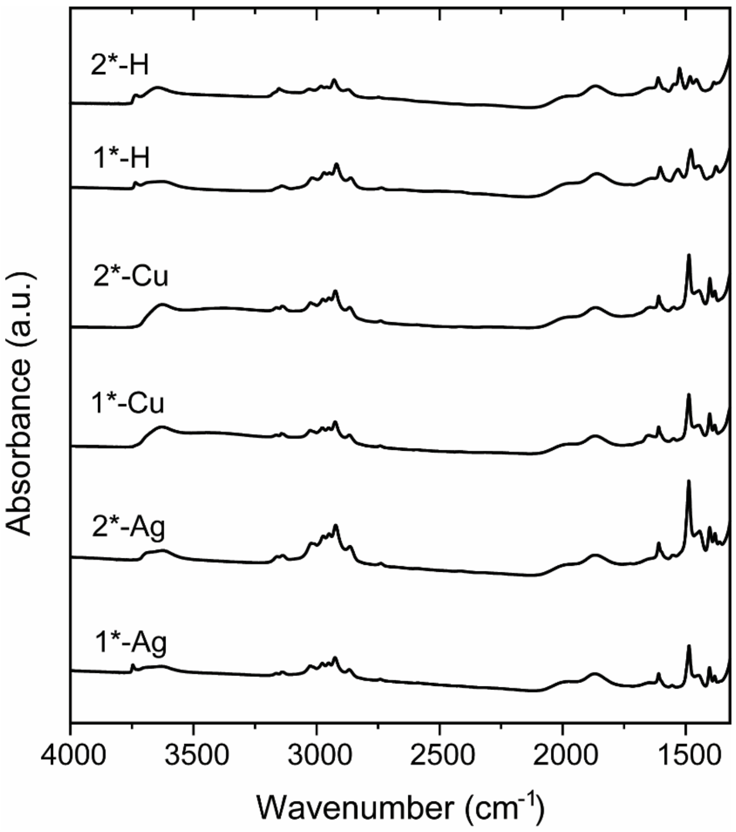

Figure S9. FTIR spectra of **1*-Ag**, **2*-Ag**, **1*-Cu**, **2*-Cu**, **1*-H**, **2*-H**.

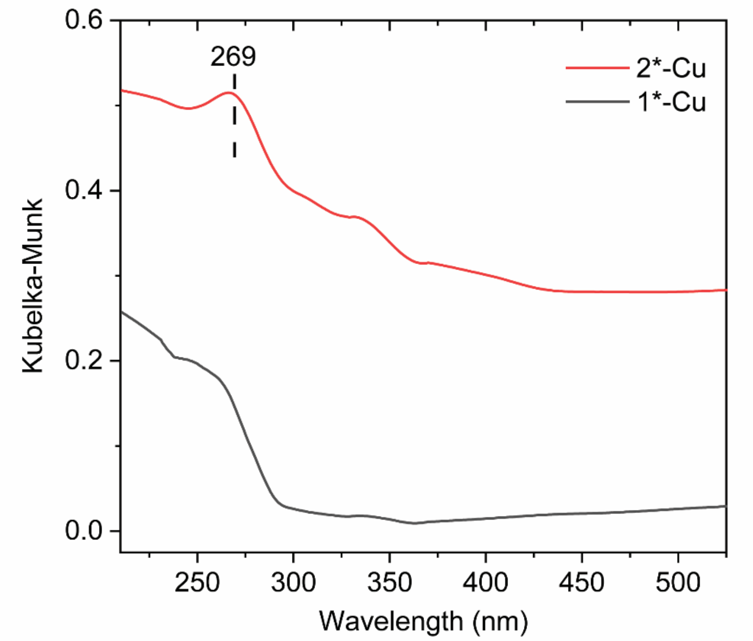

Figure S10. DRUV spectra of **1*-Cu** and **2*-Cu**.

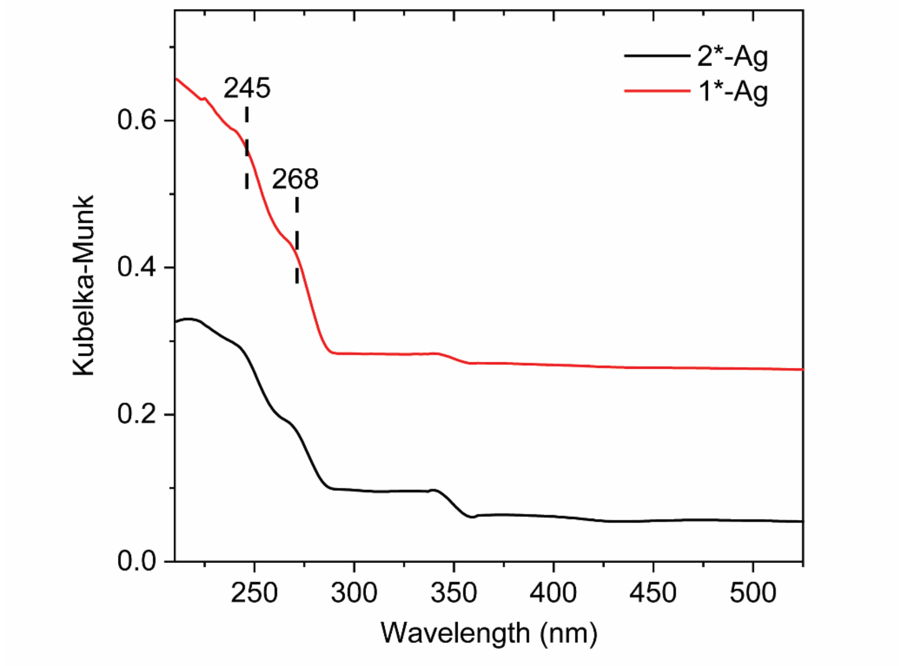

Figure S11. DRUV spectra of **1*-Ag** and **2*-Ag**.

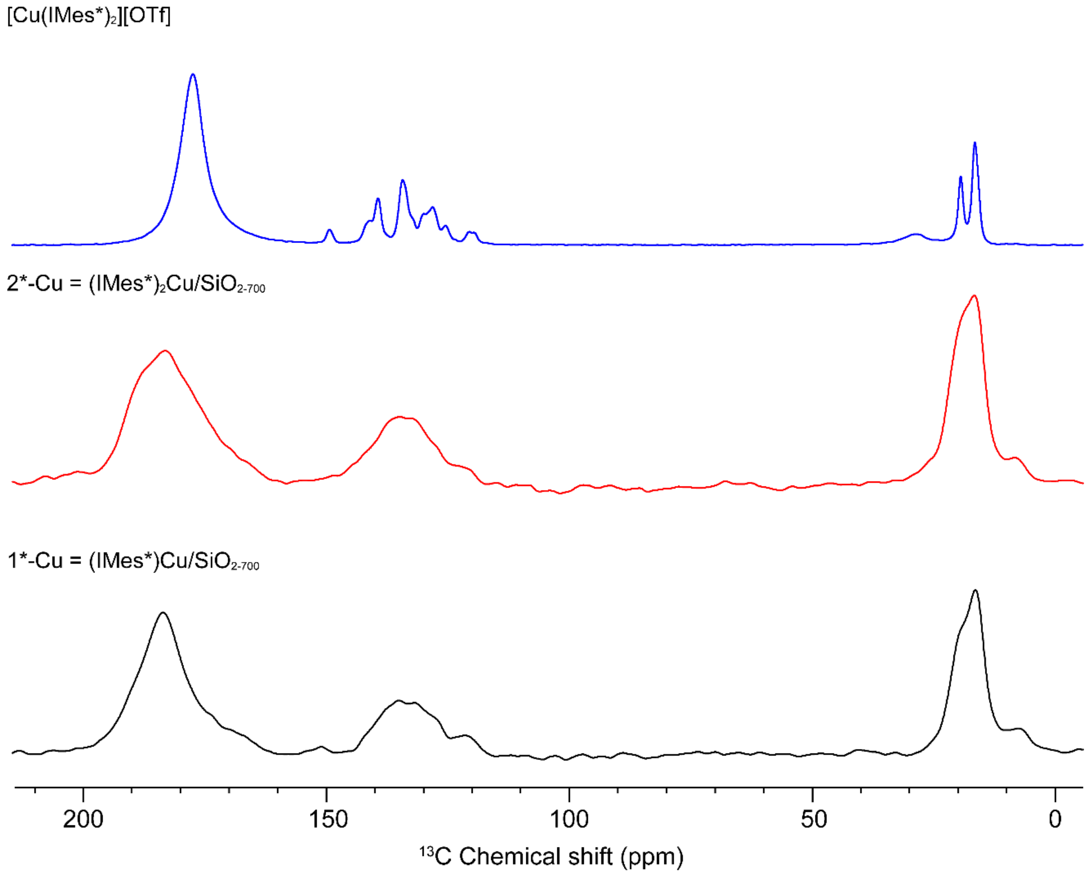

Figure S12. $^{13}C\{^{1}H\}$c CPMAS spectrum of **1*-Cu** (black), **2*-Cu** (red), $[Cu(IMes^*)_2][OTf]$ (blue). The spectra were collected at ~298 K, MAS rates of 16 kHz, and CP contact time of 2 ms.

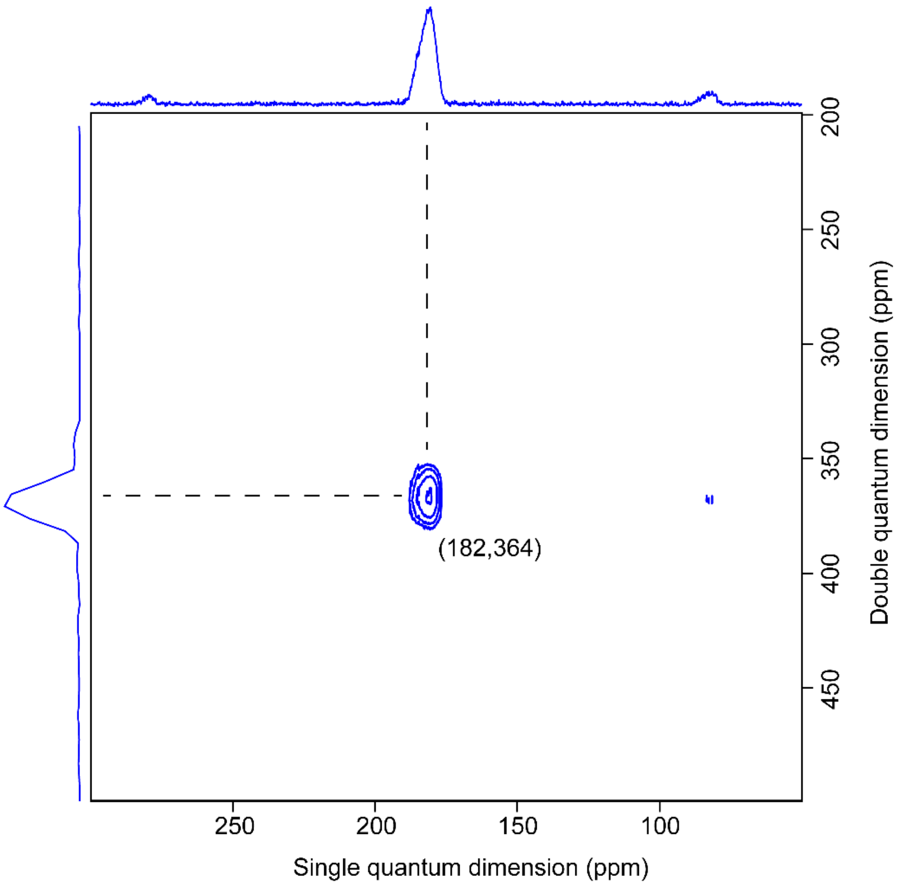

Figure S13. 2D ^{13}C INADEQUATE spectrum of $[Ag(IMes^*)_2][OTf]$, The spectra were collected at ~110 K and MAS rates of 10 kHz.

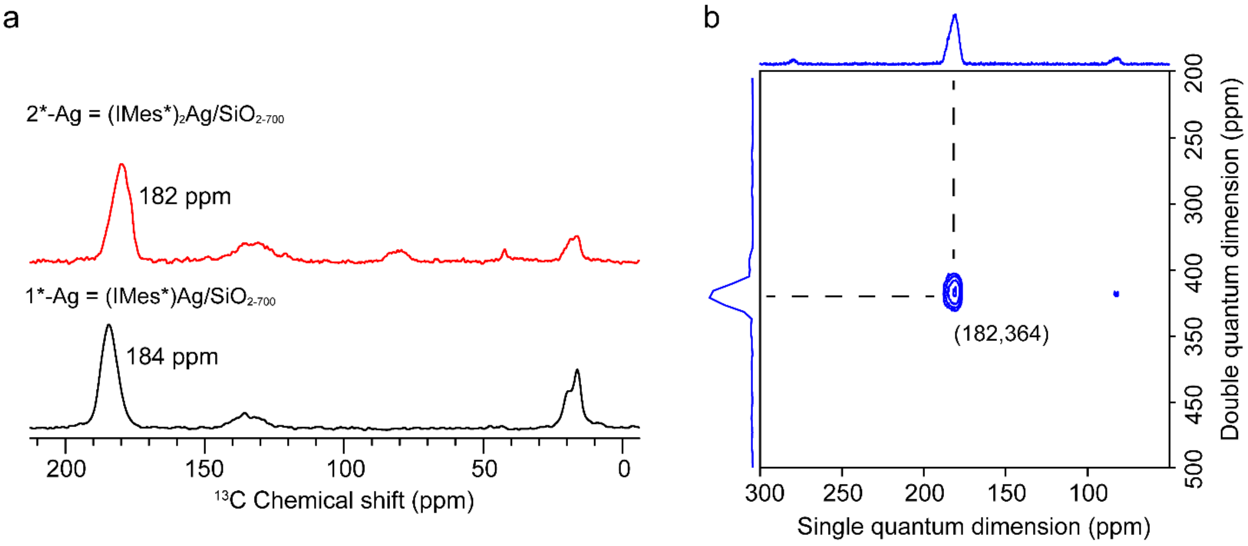

Figure S14. (a) $^{13}C\{^{1}H\}$ CPMAS spectrum of 1*-Ag (black) and 2*-Ag (red). The spectra were collected at ~298 K, MAS rates of 16 kHz, and CP contact time of 2 ms. **(b)** 2D ^{13}C INADEQUATE spectrum of 2*-Ag. The spectra were collected at ~110 K and MAS rates of 10 kHz.

Formation of AuNPs/SiO_2

SiO_{2-700} (480 mg) was impregnated with a solution of [Au_5Mes_5] (11.5 mg, 0.037 mmol) in toluene (5 mL) at room temperature. The suspension was stirred for 15 min and the solvent was evaporated in vacuo (10^{-2} mbar). After 2 h drying under high vacuum (10^{-5} mbar) a yellow solid was isolated. The physisorbed [Au_5Mes_5] was then decomposed under flowing H_2 (60 ml min^{-1} for 12 h at 300 °C, 0.5° min^{-1}) to afford a red powder (AuNPs/SiO_2). We note that Figures S15-S16 also appears in a previous reported work of our group[15].

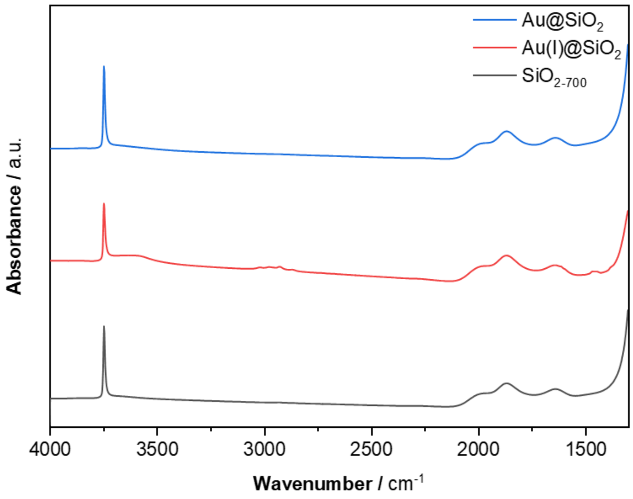

Figure S15. FTIR spectra of the different steps for AuNPs/SiO_2 synthesis.

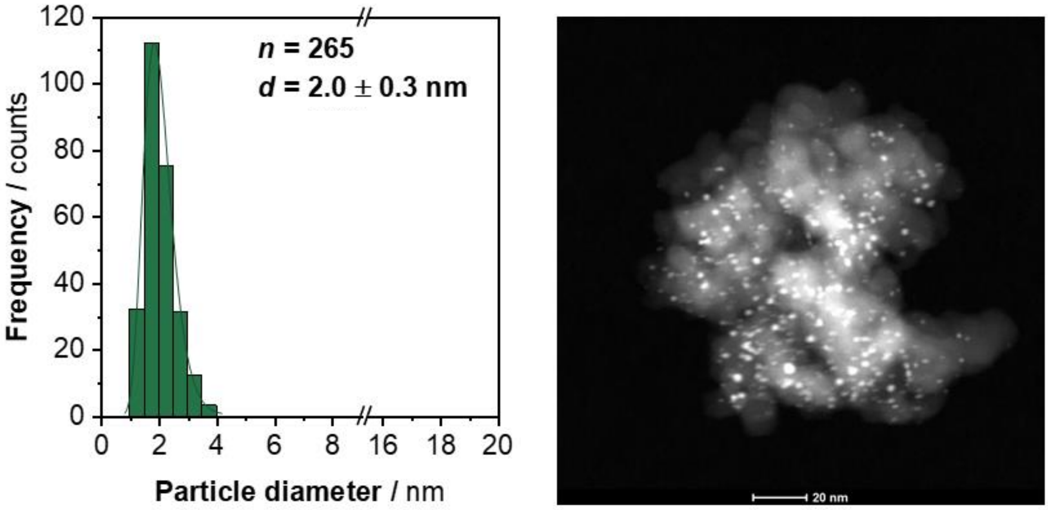

Figure S16. Size distribution analysis (left) and TEM image of AuNPs/SiO_2 (right).

IMes*-AuNPs/SiO_2

To a solution of IMes* (18 mg; 0.06 mmol) in 5 mL benzene is added Au/SiO_2 (150 mg). The mixture is stirred at 120 rpm at room temperature for 24 h. The solid is then allowed to decant and the supernatant pipetted out. The solid is then washed 3 times with 5 mL benzene, 3 time with 5 ml pentane, dried under vacuum, then high vacuum for 30 min to afford a red powder.

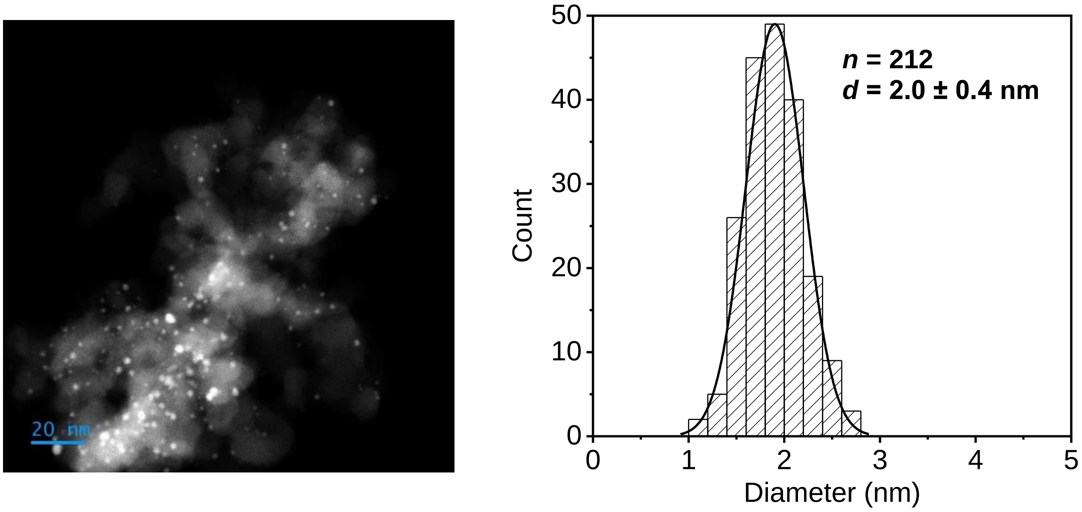

Figure S17. TEM image of IMes*-AuNPs/SiO_2 (left) and size distribution analysis (right).

XRD Studies

2.1 Crystal structures

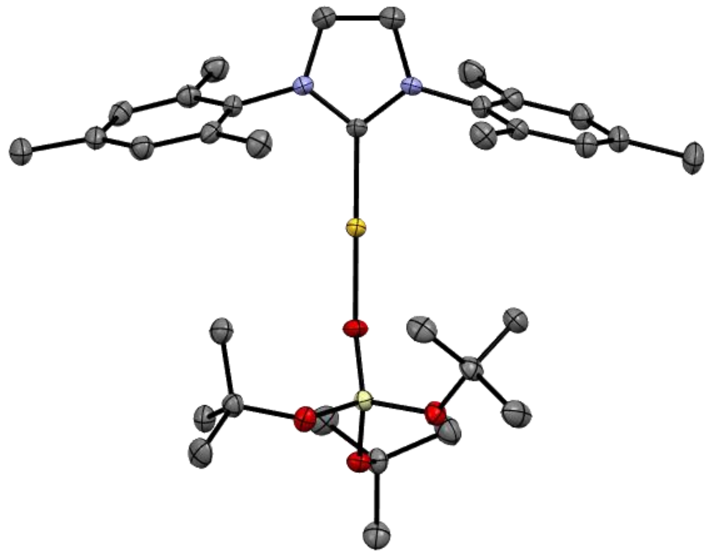

Figure S18. Crystal structure for **[Au(IMes)(OTBOS)]**. Ellipsoids shown at 50% probability. Hydrogens omitted for clarity.

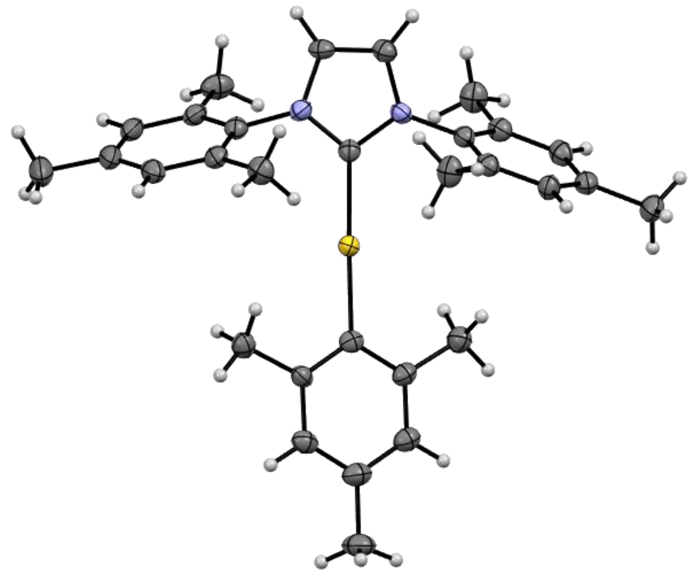

Figure S19. Crystal structure for **[Au(IMes)(Mes)]**. Ellipsoids shown at 50% probability. Hydrogens omitted for clarity.

2.2 XRD Reports

The following pages contain detailed crystallographic information on the newly synthesized molecules.

Table 1. Crystal data and structure refinement for [Au(IMes)(Mes)].

Identification code	[Au(IMes)(Mes)]
Empirical formula	$C_{30}H_{35}AuN_2$
Formula weight	620.56
Temperature/K	100(2)
Crystal system	monoclinic
Space group	$P2_1/c$
a/Å	12.0269(3)
b/Å	15.1022(4)
c/Å	14.5911(3)
α/°	90
β/°	94.661(2)
γ/°	90
Volume/Å^3	2641.46(11)
Z	4
ρ_{calc}g/cm^3	1.560
μ/mm^{-1}	10.595
F(000)	1232.0
Crystal size/mm^3	0.12 × 0.08 × 0.07
Radiation	Cu Kα (λ = 1.54184)
2Θ range for data collection/°	7.374 to 160.178
Index ranges	$-15 \leq h \leq 15$, $-19 \leq k \leq 16$, $-18 \leq l \leq 13$
Reflections collected	22922
Independent reflections	5659 [R_{int} = 0.0384, R_{sigma} = 0.0298]
Data/restraints/parameters	5659/0/307
Goodness-of-fit on F^2	1.179
Final R indexes [I>=2σ (I)]	R_1 = 0.0316, wR_2 = 0.0942
Final R indexes [all data]	R_1 = 0.0341, wR_2 = 0.0961
Largest diff. peak/hole / e Å^{-3}	1.40/-1.49

Table 2. Crystal data and structure refinement for [Au(IMes)(OTBOS)].

Identification code	[Au(IMes)(OTBOS)]
Empirical formula	C33H51AuN2O4Si
Formula weight	764.81
Temperature/K	100.00(10)
Crystal system	monoclinic
Space group	P21/n
a/Å	11.0293(3)
b/Å	16.6298(3)
c/Å	19.2398(5)
α/°	90
β/°	104.573(2)
γ/°	90
Volume/Å3	3415.34(15)
Z	4
ρcalcg/cm3	1.487
μ/mm 1	8.717
F(000)	1552.0
Crystal size/mm3	0.28 × 0.16 × 0.14
Radiation	Cu Kα (λ = 1.54184)
2Θ range for data collection/°	7.126 to 161.372
Index ranges	-14 ≤ h ≤ 14, -21 ≤ k ≤ 19, -24 ≤ l ≤ 24
Reflections collected	101433
Independent reflections	7464 [Rint = 0.0375, Rsigma = 0.0133]
Data/restraints/parameters	7464/0/386
Goodness-of-fit on F2	1.198
Final R indexes [I>=2σ (I)]	R1 = 0.0338, wR2 = 0.1044
Final R indexes [all data]	R1 = 0.0342, wR2 = 0.1049
Largest diff. peak/hole / e Å-3	1.83/-1.60

3. References

1. Jafarpour, L.; Stevens, E. D.; Nolan, S. P., A sterically demanding nucleophilic carbene: 1,3-bis(2,6-diisopropylphenyl)imidazol-2-ylidene). Thermochemistry and catalytic application in olefin metathesis. *J. Organomet. Chem.* **2000,** *606* (1), 49-54.
2. Siddiqi, G.; Mougel, V.; Copéret, C., [Au5Mes5]: improved gram-scale synthesis and its use as a convenient precursor for halide-free supported gold nanoparticles. *Dalton Trans.* **2015,** *44* (32), 14349-14353.
3. De Frémont, P.; Scott, N. M.; Stevens, E. D.; Nolan, S. P. J. O., Synthesis and structural characterization of N-heterocyclic carbene gold (I) complexes. *Organometallics* **2005,** *24* (10), 2411-2418.
4. Lozada-Rodríguez, L.; Pelayo-Vázquez, J. B.; Rangel-Salas, I. I.; Alvarado-Rodríguez, J. G.; Peregrina-Lucano, A. A.; Pérez-Centeno, A.; López-Dellamary-Toral, F. A.; Cortes-Llamas, S. A., From metallic gold to [Au(NHC)2]+ complexes: an easy, one-pot method. *Dalton Trans.* **2017,** *46* (12), 3809-3811.
5. Hansen, C.; Docherty, S.; Cao, W.; Yakimov, A.; Copéret, C., 109Ag NMR Chemical Shift as a Descriptor for Brønsted Acidity from Molecules to Materials. *ChemRxiv* **2023**.
6. Kaeffer, N.; Mance, D.; Copéret, C., N-Heterocyclic Carbene Coordination to Surface Copper Sites in Selective Semihydrogenation Catalysts from Solid-State NMR Spectroscopy. *Angew. Chem. Int. Ed.* **2020,** *132* (45), 20174-20182.
7. Arduengo, A. J., III; Dias, H. V. R.; Calabrese, J. C.; Davidson, F., Homoleptic carbene-silver(I) and carbene-copper(I) complexes. *Organometallics* **1993,** *12* (9), 3405-3409.
8. Fulmer, G. R.; Miller, A. J. M.; Sherden, N. H.; Gottlieb, H. E.; Nudelman, A.; Stoltz, B. M.; Bercaw, J. E.; Goldberg, K. I., NMR Chemical Shifts of Trace Impurities: Common Laboratory Solvents, Organics, and Gases in Deuterated Solvents Relevant to the Organometallic Chemist. *Organometallics* **2010,** *29* (9), 2176-2179.
9. Dolomanov, O. V.; Bourhis, L. J.; Gildea, R. J.; Howard, J. A. K.; Puschmann, H., OLEX2: a complete structure solution, refinement and analysis program. *J. Appl. Crystallogr* **2009,** *42* (2), 339-341.
10. Palatinus, L.; Prathapa, S. J.; van Smaalen, S., EDMA: a computer program for topological analysis of discrete electron densities. *J. Appl. Crystallogr* **2012,** *45* (3), 575-580.
11. Sheldrick, G., SHELXT - Integrated space-group and crystal-structure determination. *Acta Crystallogr. A* **2015,** *71* (1), 3-8.
12. Lesage, A.; Bardet, M.; Emsley, L., Through-Bond Carbon−Carbon Connectivities in Disordered Solids by NMR. *J. Am. Chem. Soc.* **1999,** *121* (47), 10987-10993.
13. Bain, A. D.; Hughes, D. W.; Anand, C. K.; Nie, Z.; Robertson, V. J., Problems, artifacts and solutions in the INADEQUATE NMR experiment. *Magn. Reson. Chem.* **2010,** *48* (8), 630-641.
14. Bain, A. D., Modulation of NMR spin echoes in coupled systems. *Chem. Phys. Lett.* **1978,** *57* (2), 281-284.
15. Docherty, S. R.; Safonova, O. V.; Copéret, C., Surface Redox Dynamics in Gold–Zinc CO2 Hydrogenation Catalysts. *J. Am. Chem. Soc.* **2023,** *145* (25), 13526-13530.
